# *Codonopsis pilosula* polysaccharide attenuates Aβ toxicity and cognitive defects in APP/PS1 mice

**DOI:** 10.18632/aging.103445

**Published:** 2020-07-11

**Authors:** Lu Wan, Qing Zhang, Hongbin Luo, Zhendong Xu, Sheng Huang, Fumin Yang, Yi Liu, Yacoubou Abdoul Razak Mahaman, Dan Ke, Qun Wang, Rong Liu, Jian-Zhi Wang, Xiji Shu, Xiaochuan Wang

**Affiliations:** 1 Department of Pathophysiology, School of Basic Medicine, Key Laboratory of Education Ministry of China for Neurological Disorders, Tongji Medical College, Huazhong University of Science and Technology, Wuhan 430030, China; 2 Medical College, Hubei University for Nationalities, Enshi 445000, HB, China; 3 Co-innovation Center of Neuroregeneration, Nantong University, Nantong 226001, JS, China; 4 Department of Pathology and Pathophysiology, School of Medicine, Jianghan University, Wuhan 430056, China

**Keywords:** Alzheimer’s disease, polysaccharide *Codonopsis pilosula*, BACE1, Aβ, cognitive impairment

## Abstract

*Codonopsis pilosula* Polysaccharides (CPPs), a traditional Chinese medicine used for thousands of years, is a potential neuroprotective polysaccharide via a relatively poorly understood mechanism. We previously reported that CPPs attenuated tau pathology in hTau transfected mice and therefore in the current work investigated the effect of CPPs on Aβ toxicity and cognitive defects in APP/PS1 mice model. It was found that one-month intragastric administration of CPPs significantly ameliorated cognitive defects in APP/PS1 mice. In addition, CPPs treatment mitigated the loss of the synaptic plasticity and increased the synaptic proteins including synaptotagmin and PSD95. The expression of Aβ42 and Aβ40 was remarkably decreased in the hippocampus of APP/PS1 mice after CPPs treatment. We also found that CPPs coincubation significantly reduced the amount of APPβ and Aβ42 expression in cells. Intriguingly, the activity of BACE1 was decreased following CPPs treatment in both the hippocampus of APP/PS1 mice and in vitro experiments. Collectively, these results indicated that CPPs attenuated Aβ pathology in APP/PS1 mice, and down-regulating BACE1 might be the underlaying mechanism which could be a therapeutic target for alleviating cognitive defects in AD pathology.

## INTRODUCTION

Alzheimer’s disease (AD) is a neurodegenerative disease characterized by memory loss and progressive cognitive impairment, and it represents the most common form of dementia [1, 2]. Currently, the global prevalence of dementia is about 47 million, and the number is predicted to be more than tripled by 2050 [3]. From a histopathological point of view, AD is revealed by the presence of senile plaques (SP) comprised predominantly of amyloid-β (Aβ) peptides and neurofibrillary tangles (NFTs) made of hyperphosphorylated tau [4, 5].

An imbalance between the production and clearance of Aβ42 and related Aβ peptides leads to abnormal accumulation of extracellular Aβ [6]. It has been reported that Aβ application induced long-term synaptic depression and memory impairment in rodent hippocampus [7, 8]. Moreover, exposure to Aβ42 also triggered hyperphosphorylation of tau at AD-relevant epitopes in cultured neurons [9]. Beta-site APP cleaving enzyme 1 (BACE1), a single transmembrane aspartyl-protease, which is responsible for the cleavage of amyloid precursor protein (APP) to produce APPβ, is the rate limiting enzyme in the amyloidogenic APP processing and thus plays an important role in Aβ generation [10, 11]. Therefore, inhibition of BACE1 has become a promising strategy to reduce the Aβ toxicity in the AD therapeutics [12].

As the etiology of AD is multifactorial, natural products which usually contain numerous ingredients with synergic effect would be beneficial for the treatment of the condition [13, 14]. Natural products have been used in many countries for the treatment of various diseases, for their bioavailable peculiarity and relatively less side-effects [15]. *Codonopsis pilosula* (CP) is a kind of Chinese herbal medicine with long history, which has complex component including polysaccharides, sesquiterpenes, saponins, polyphenolic glycosides, polyacetylenes, alkaloids, essential oils, and phytosterols [16–18]. The CP polysaccharides (CPPs) are active compounds extracted from CP, which have been identified to possess multiple pharmacological functions such as antitumor, antimicrobial, antioxidant, and immunoenhancing properties [19–22]. It has been reported that heparan sulfate polysaccharides interact with BACE1 and regulate its APP cleaving activity, mainly by blocking access of substrate to the active site [23]. Previous studies have also provided evidence that natural polysaccharides mitigated cognitive deficits in animal models of AD [24–27]. However, whether CPPs alleviate AD pathological process, especially as anti-Aβ accumulation is yet to be known.

Our previous work showed that CPPs attenuated tau hyperphosphorylation and cognitive impairments in hTau transfected mice [28]. In the current study, we found that CPPs significantly ameliorated cognitive defects in APP/PS1 mice after one-month intragastric administration. In addition, coincubation of CPPs with BACE1 in cultured cells inhibited BACE1 activity *in vitro*. Hence, our findings indicated that CPPs-mediated down-regulation of BACE1 activity might be a potential therapeutic strategy for mitigating cognitive deficits in AD.

## RESULTS

### CPPs alleviated cognitive impairments in APP/PS1 mice

**Figure 1:**
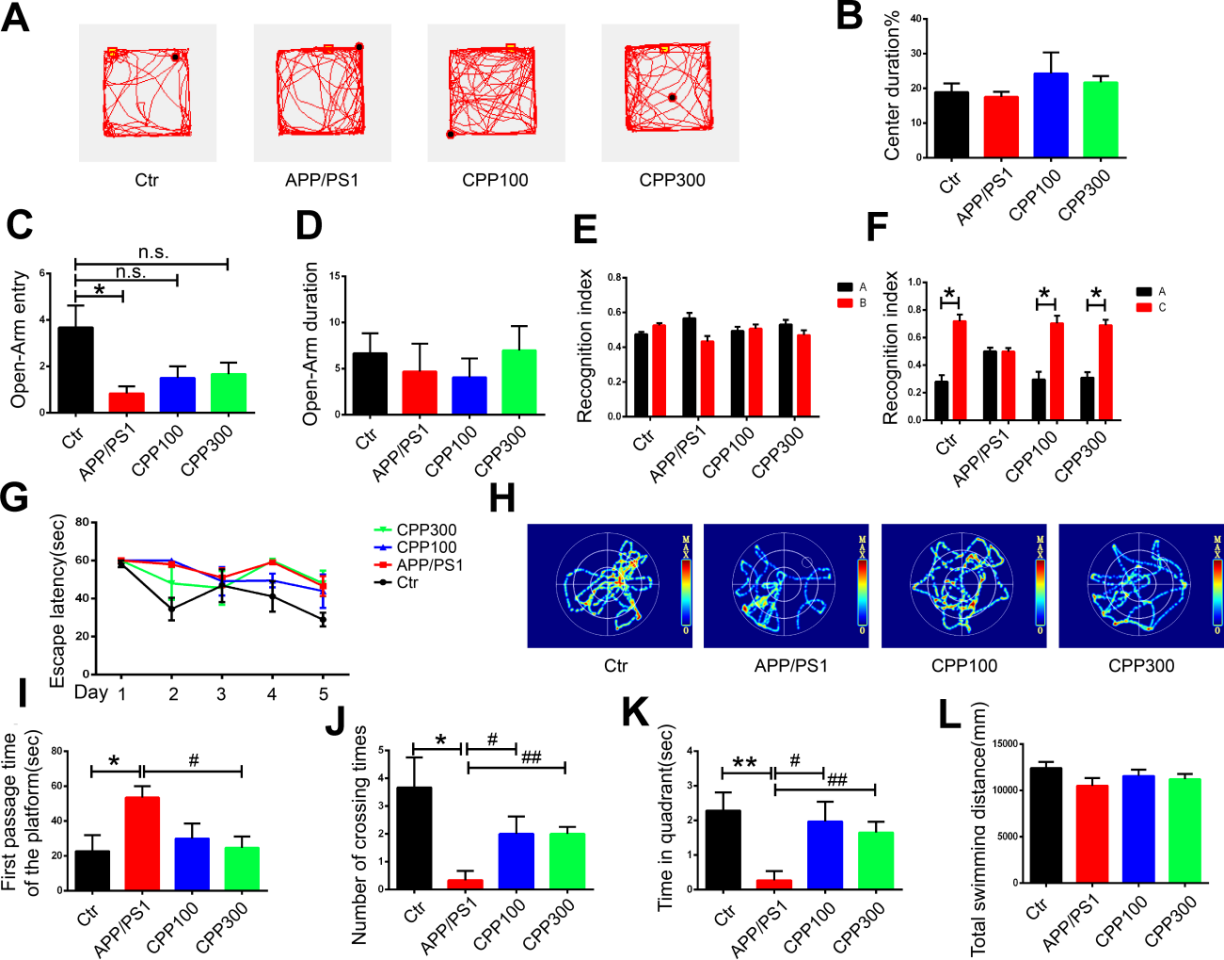
**CPPs alleviated cognitive impairments in APP/PS1 mice.** 5.5-month-old C57/BL6 mice served as WT control and were treated with IG administration of normal saline for one month. 5.5-month-old APP/PS1 mice were treated with one-month IG administration of 100 or 300 mg/kg of CPPs, or with normal saline as positive control. At the age of 6.5 month, all the mice were assessed in behavioral tests. (**A**–**D**) Open field tests and Elevated plus maze tests were used to assess the anxiety-like behavior of mice. (**A**) Representative trajectory, (**B**) Center duration, (**C**) Open-Arm entry, (**D**) Open-Arm duration. (**E**, **F**) NOR experiment was performed to assess the recognition index. (**E**) Recognition index between object A and B were detected. Recognition index were recorded in 5 min. After 24 h, object C was used instead of object B. (**F**) Recognition index between object A and C were detected. (**G**–**L**) Morris water maze (MWM) test was performed to evaluate the spatial memory of mice. (**G**) Escape latency of during the 5 days trial. (**H**) Representative trajectory of mice in the probe trial. (**I**) The first passage time of the platform in the target quadrant in 60 sec after removing the platform. (**J**) The number of crossing times. (**K**) The time spent in target quadrant. (**L**) The total swimming distance of each group. P value significance is calculated from a one-way ANOVA or two-way ANOVA. *P < 0.05, **P < 0.01, vs. WT control mice, #P < 0.05, ##P < 0.01 vs. positive control (APP/PS1 mice without CPPs treatment), n = 6.

Alterations in behavioral and cognitive functions, including learning and memory, are one of the characteristic features of AD [29]. To explore whether CPPs can attenuate behavioral impairments in the APP/PS1 AD mice model, we assessed the behavioral changes in 6.5-month-old wild-type (WT) and APP/PS1 mice, with or without the one-month daily intragastric (IG) administration of 100 and 300 mg/kg of CPPs, when normal saline served as vehicle control. At the age of 6.5 month, we first performed open field test and elevated plus maze test to detect the anxiety-like behavior in the mice. There was no significant difference in center duration among all groups in the open field test (Figure 1A, 1B). However, in the elevated plus maze test a significant reduction in the open-Arm entries was observed in APP/PS1 mice compared with WT mice, whereas there was an upward trend in the 100 or 300 mg/kg CPPs treated APP/PS1 mice with no statistical difference as compared with the control WT mice (Figure 1C). On the other hand, no obvious difference in open-Arm duration was observed among all the groups (Figure 1D). These findings indicated that CPPs treatment might attenuate anxiety-like behavior of APP/PS1 mice.

At the early stage of AD, learning and memory decline, a hippocampus-dependent cognitive function, has been reported [30]. We used the Novel Objective Recognition (NOR) Test and Morris Water Maze (MWM) test to investigate the effects of CPPs on the hippocampus-dependent spatial learning and memory of mice. In the acquisition trial in NOR test, no significant difference in the recognition index was detected among all the groups (Figure 1E). Interestingly, during the test trials, 24h following acquisition, we found that CPPs treatment increased the recognition index toward the new objective compared with normal saline treated APP/PS1 mice, suggesting potential effects of CPPs treatment on long-term memory repair (Figure 1F). Further, we performed the MWM test where the mice were trained for five consecutive days with three trials per day and found that there were no statistic differences between CPP-treated APP/PS1 and control APP/PS1 mice, suggesting that CPP might don’t affect learning ability (Figure 1G). On the seventh day, the probe trial was carried out without platform (Figure 1H). There was a conspicuous increase in the first passage time and a decrease in the number of crossing times of the APP/PS1 mice compared with control (Figure 1I–1L). However, treatment of CPPs with 300 mg/kg but not 100 mg/kg restored the first passage time of APP/PS1 mice to control level (Figure 1I). Moreover, number of crossing the position of the platform and time spent in the target quadrant were rescued in CPPs treatment groups compared with APP/PS1 treated with normal saline group (Figure 1J, 1K). The motor activity, as reflected by the total swimming distance, showed no significant difference among all groups (Figure 1L). Taken together, these results clearly indicated that CPPs treatment significantly attenuated cognitive deficits in APP/PS1 mice.

### CPPs recovered synaptic plasticity alterations in APP/PS1 mice

**Figure 2:**
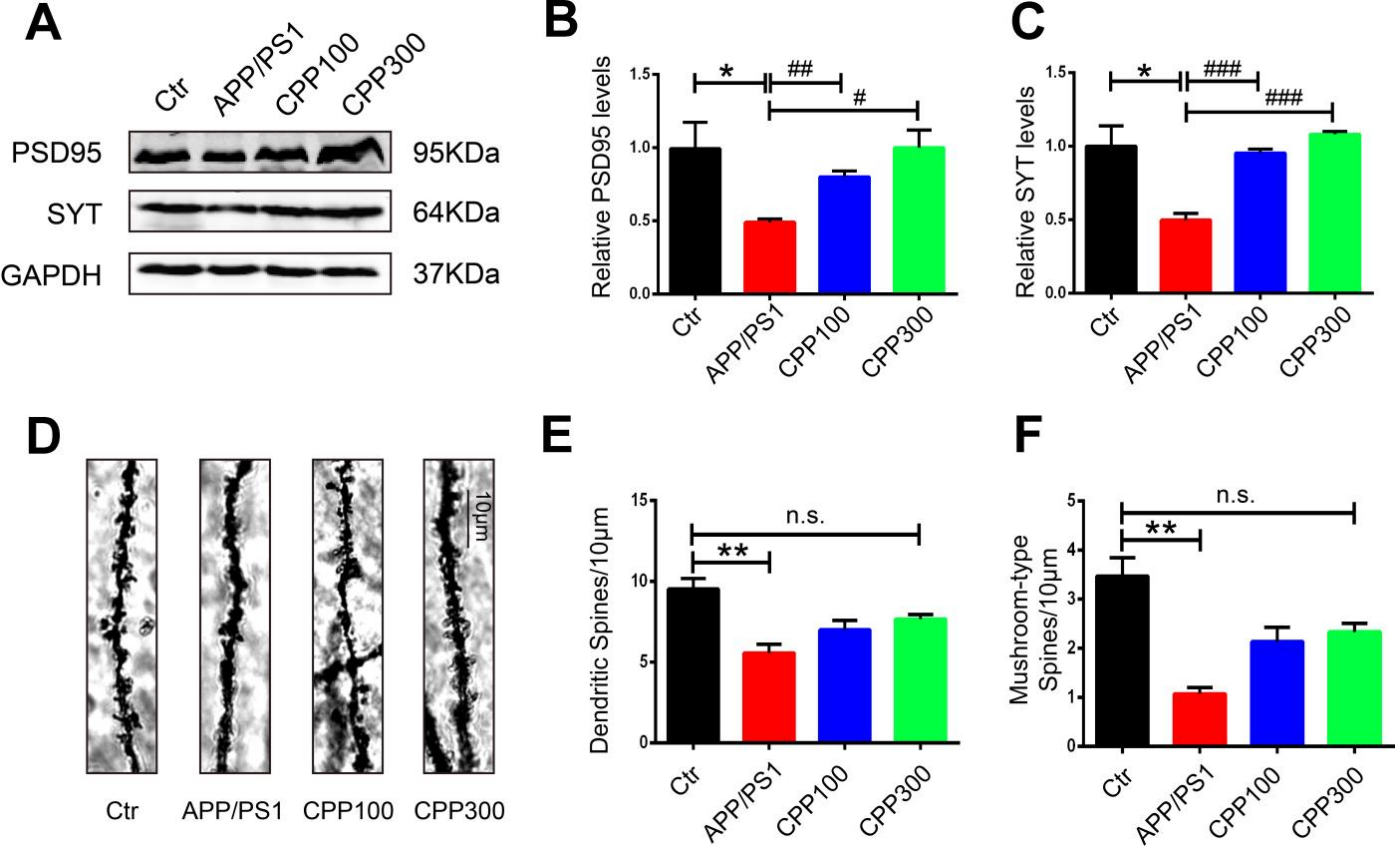
**CPPs recovered the loss of synaptic proteins and synaptic plasticity in APP/PS1 mice.** Synaptic proteins and spine density were evaluated in APP/PS1 mice after IG administration of 100, 300 mg/kg of CPPs or saline as control for one month. (**A**) Levels of PSD95, and synaptotagmin (SYT) were detected by western blotting in the hippocampus and GAPDH was used as loading control. (**B**, **C**) Quantitative analysis of the blots. (**D**) Representative Golgi staining images. (**E**) The quantification of spine density and (**F**) Mushroom type spine. P value significance is calculated from a one-way ANOVA. The data were expressed as mean ± SEM, *P < 0.05, **P < 0.01 vs. WT mice, #P < 0.05, ##P < 0.01, ###P < 0.001 vs. positive control (APP/PS1 mice without CPPs treatment), n = 3.

Learning and memory efficiency are a reflection of normal synaptic function and are integrated via synaptic plasticity [31]. To further investigate how CPPs treatment attenuated cognitive deficits and whether it affected synaptic plasticity in APP/PS1 mice, the expression of PSD95 and synaptotagmin was evaluated in the hippocampal homogenate by western blot in all groups. The results showed that administration of CPPs significantly increased the expression of PSD95 and synaptotagmin levels, in comparison to APP/PS1 mice without CPPs treatment (Figure 2A–2C). To further investigate synaptic integrity which are the basis of learning and memory, Golgi staining was carried out to evaluate the dendritic spines in the hippocampus of mice brain. Notably, significant reduction of dendritic spines and mushroom type spines was observed in APP/PS1 mice compared with the WT mice (Figure 2D–2F). Unlike the normal saline treated APP/PS1 mice, there were no significant differences in dendritic spines and mushroom type spines in the hippocampus of 300 mg/kg treated APP/PS1 mice compared with the WT mice (Figure 2D–2F). These results indicated that CPPs might ameliorate cognitive defects through the restoration of synaptic plasticity. Altogether these findings suggested that CPPs treatment had a potential pharmacological role in recovering the synaptogenesis and synaptic plasticity.

### CPPs treatment prevented Aβ accumulation in APP/PS1 mice

**Figure 3:**
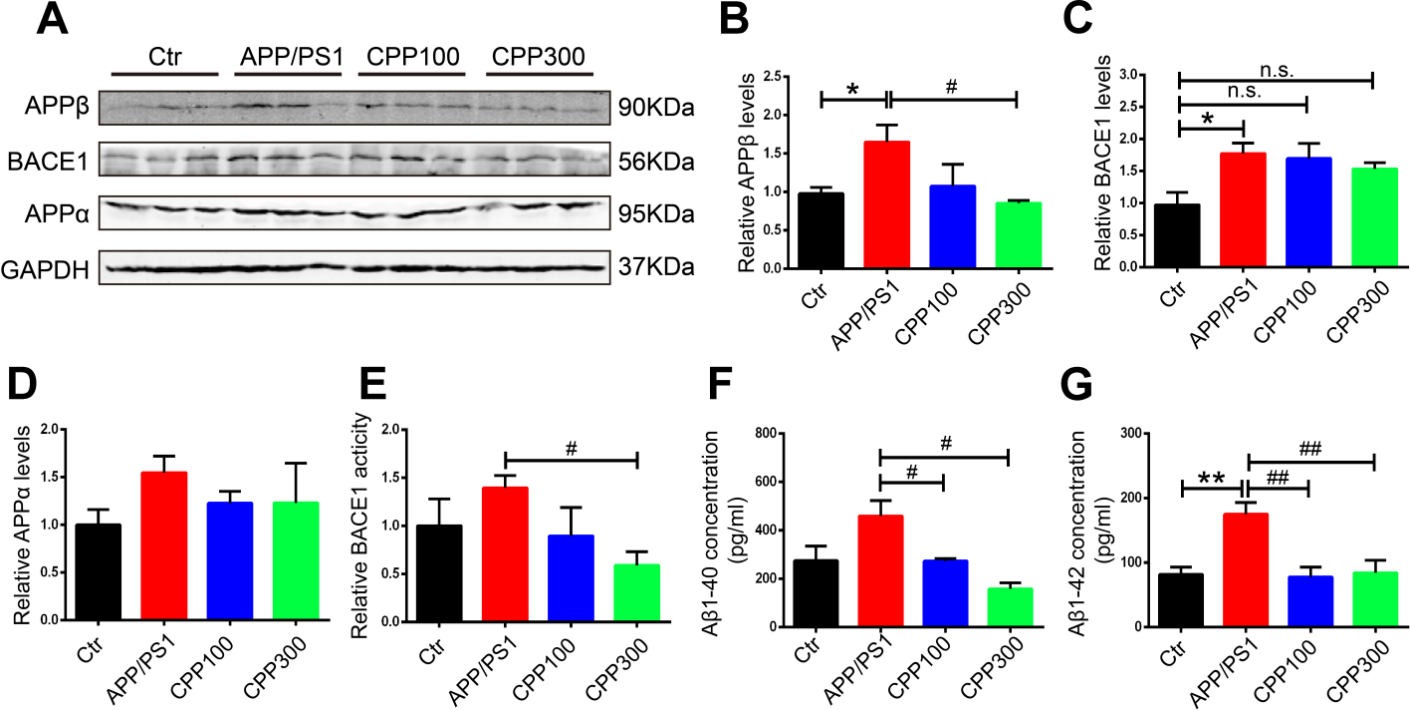
**CPPs treatment prevented Aβ accumulation in APP/PS1 mice.** (**A**) Western blotting was performed to detect the expression of APPβ, BACE1 and APPα proteins in the hippocampus. (**B**–**D**) Quantitative analysis of the blots. (**E**) Relative fluorescence levels of BACE1 were detected in the hippocampal lysate. (**F**, **G**) Hippocampal tissue homogenates were assayed for Aβ1-40 (**F**) or Aβ1-42 (**G**) levels. P value significance is calculated from a one-way ANOVA. The data were expressed as mean ± SEM, *P < 0.05, **P < 0.01 vs. WT mice, #P < 0.05, ##P < 0.01 vs. positive control (APP/PS1 mice without CPPs treatment), n = 3.

Senile plaques, the core component of which are Aβ, are one of the two hallmarks of AD and APP/PS1 mice is a typical Aβ-related AD mice model [3]. We therefore next determined whether CPPs treatment could ameliorate Aβ pathology in APP/PS1 mice. The APP proteolytic pathway was examined. The western blot result showed no significant difference in the expression of APPα in the hippocampal homogenate of all the groups, whereas the APPβ protein levels was decreased in the 300 mg/kg treated APP/PS1 mice compared with normal saline treated APP/PS1 ones (Figure 3A–3D). To further confirm this result, we evaluated the protein level of BACE1 which is the key proteolytic enzyme leading to Aβ production. Compared with the WT mice, there was an increase of BACE1 levels in APP/PS1 mice, whereas no significant differences were detected in CPPs treated APP/PS1 mice (Figure 3C). Interestingly, we also found that BACE1 activity in the hippocampus was greatly reduced in the APP/PS1 mice that received the 300 mg/kg CPPs treatment compared with normal saline treated APP/PS1 mice (Figure 3E). Besides, the ELISA result showed that CPPs treatment reduced the levels of Aβ40 and Aβ42 in the hippocampus of APP/PS1 mice compared with normal saline treated APP/PS1 mice (Figure 3F, 3G), suggesting that CPPs treatment affected Aβ production and/or accumulation. The aggregate findings, therefore, indicated that CPPs effectively attenuated the Aβ pathology in the treated animals.

### Supplementation with CPPs ameliorated Aβ pathology *in vitro*

**Figure 4:**
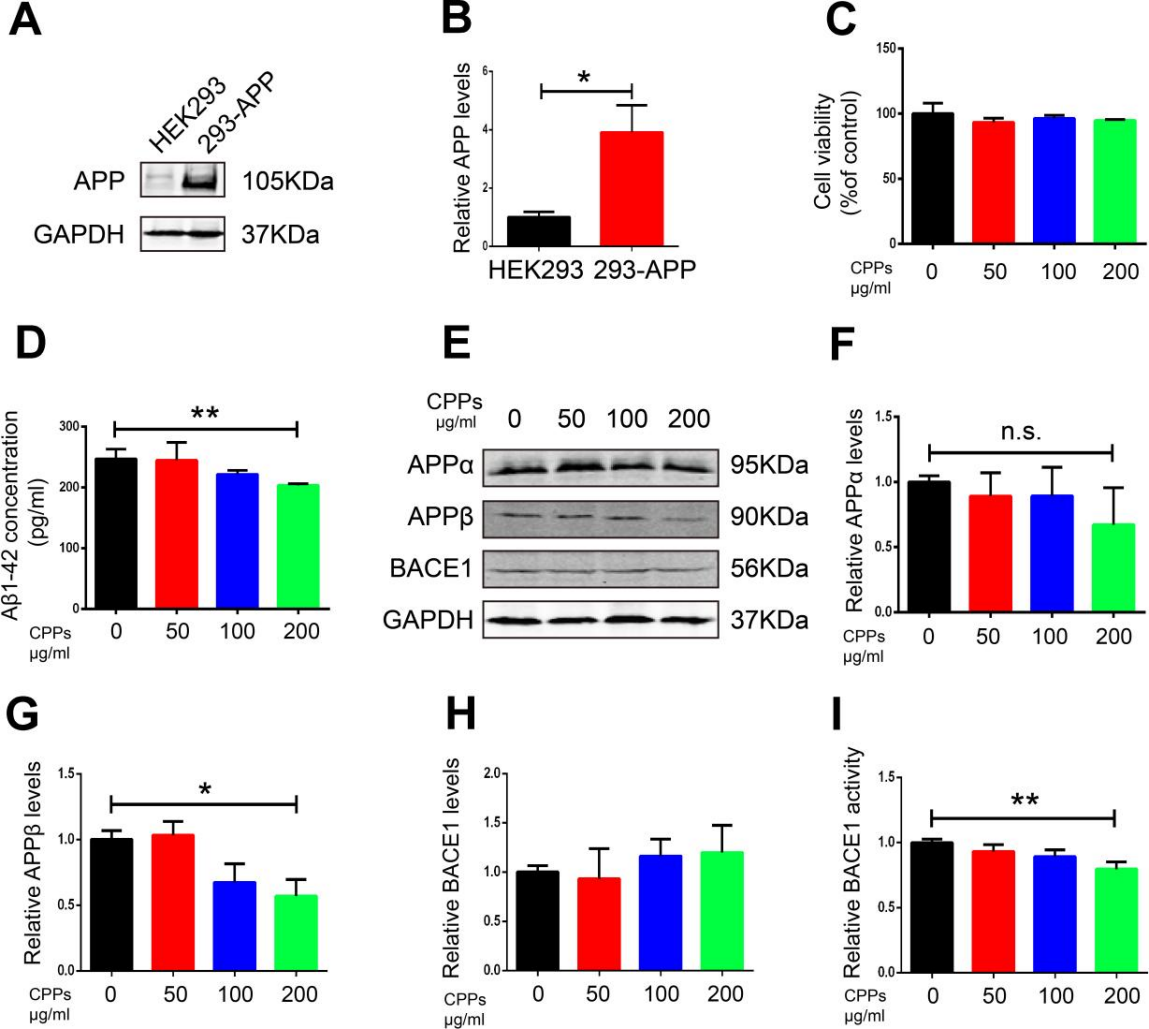
**Supplementation with CPPs mitigated Aβ pathology in APP transfected HEK293 cells.** (**A**) Western blotting was performed to detect the expression of APP in HEK293 cells transfected with APP plasmid or vector (n=3). (**B**) Quantification of the western blot results with APP normalized to GAPDH. (**C**) HEK293 cells were transfected with APP plasmid (293-APP cells) followed by CPPs supplementation at the indicated concentrations of 50, 100, 200 μg/ml for 24 h. 293-APP cells were assayed for cell viability, n = 3. (**D**) ELISA assay for Aβ1-42 levels, n = 3. (**E**–**H**) The expression of APPα, APPβ and BACE1 proteins in the 293-APP cells was measured by Western blotting, normalized to GAPDH, n = 3. (**I**) Relative BACE1 activity was detected in the cells (n = 6). P value significance is calculated from a one-way ANOVA or Student’s t-test. *P < 0.05, **P < 0.01 vs. cells without CPPs treatment.

**Figure 5:**
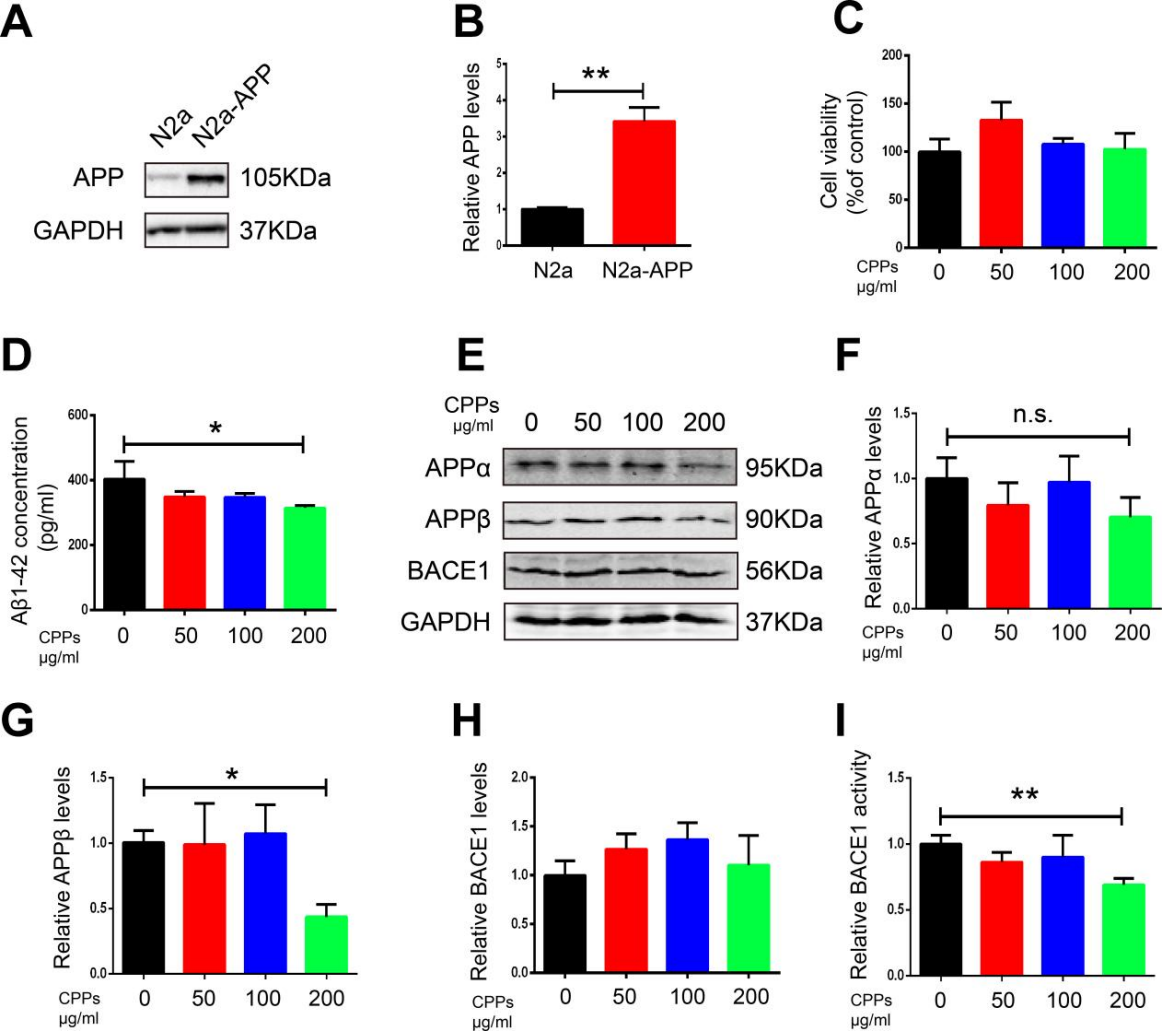
**Supplementation with CPPs ameliorated Aβ pathology in N2a-APP cells.** (**A**)The expression of APP in N2a-APP cells was estimated by western blot compared with N2a cells(n=3). (**B**) Quantification of the western blot results with APP normalized to GAPDH. N2a-APP cells were treated with CPPs at the indicated concentrations of 50, 100, 200 μg/ml for 24 h. (**C**) N2a-APP cells were assayed for cell viability, n = 3. (**D**) ELISA assay for Aβ1-42 levels, n = 3. (**E**–**H**) The expression of APPα, APPβ and BACE1 proteins in the N2a-APP cells was measured by Western blotting, normalized to GAPDH, n = 3. (**I**) Relative BACE1 activity was detected in the cells (n=6). P value significance is calculated from a one-way ANOVA or Student’s t-test. *P < 0.05, **P < 0.01 vs. cells without CPPs treatment.

To further confirm the effect of CPPs on Aβ pathology, we assessed the effects of CPPs treatment on two cellular models. We incubated N2a-APP cells or HEK293 cells transfected with APP plasmid (293-APP) with different concentration of CPPs. First, we pretreated HEK293 cells with APP plasmid or vector, followed by CPPs treatment for 24h. As expected, 293-APP cells demonstrated significantly higher expression of APP compared with HEK293 cells (Figure 4A, 4B). Similarly, APP expression in N2a-APP cells and N2a cells was texted by western blot (Figure 5A, 5B). CCK8 cell viability assay kit was employed to evaluate the viability of cells, the results showed that CPPs had no adverse effects on cell viability in 293-APP cells (Figure 4C) and N2a-APP cells (Figure 5C) even at a high concentration as 200 μg/ml. We then evaluated Aβ1-42 level by ELISA assay, and the result determined a significantly lower levels of Aβ1-42 in 200μg/ml CPPs treated 293-APP cells (Figure 4D) and N2a-APP cells (Figure 5D) compared with the control. To further investigate the effects of CPPs on Aβ generation, the expression of Aβ production related proteins was monitored. We found that although the level of APPα was decreased following 200μg/ml CPPs treatment it does not reach significance (Figure 4E, 4F and Figure 5E, 5F), however the protein level of APPβ was significantly reduced in CPPs treated cells (Figure 4E, 4G and Figure 5E, 5G) compared with the control. We also evaluated the expression and activity of BACE1 and the result showed no differences in the expression but a significant reduction in the activity of BACE1 in 200 μg/ml CPPs treated cells (Figure 4E, 4H, 4I and Figure 5E, 5H, 5I), further indicating that CPPs might down-regulate BACE1 activity to prevent Aβ accumulation.

### CPPs relieved Aβ toxicity in rat primary neuron

**Figure 6:**
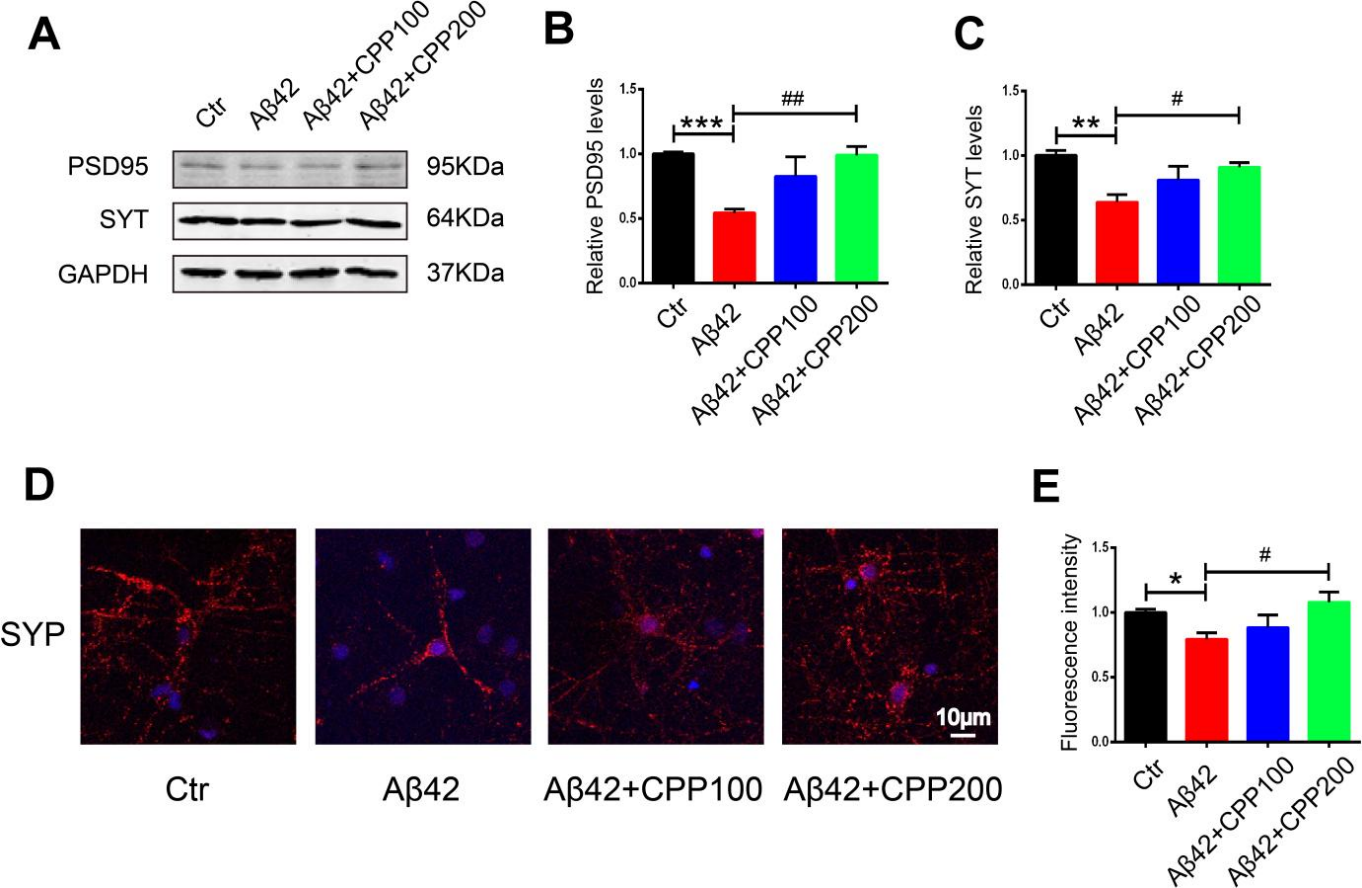
**CPPs relieved Aβ-induced toxicity in rat primary neuron in vitro.** On exposure to pre-aggregating Aβ1–42 or vehicle for 24h, rat primary neuron was treated with or without CPPs at the indicated concentrations of 100, 200 μg/ml for 24 h. (**A**) The expression of PSD95 and synaptotagmin (SYT) proteins in the primary neuron were detected by Western blotting, GAPDH as a loading control. (**B**, **C**) Quantitative analysis of the blots, n = 3. (**D**) Immunofluorescence staining was used to measure the expression of SYP in primary neuron. (**E**) Quantitative analysis of fluorescence intensity. Data were from 3 independent experiments. P value significance is calculated from a one-way ANOVA. **P < 0.01, ***P < 0.001 vs. control, #P < 0.05, ##P < 0.01vs. cells with Aβ treatment but without CPPs treatment, n = 3.

Numerous studies have reported the synaptotoxicity of Aβ in neurons [32, 33]. We therefore next explored whether CPPs treatment has a potential effect to ameliorate Aβ toxicity in rat primary neuron. The results as evaluated by western blot showed that treatment with 200μg/ml CPPs significantly increased the expression of PSD95 and synaptotagmin levels in primary neuron pretreated with aggregating Aβ1-42 (Figure 6A–6C). Moreover, immunofluorescence staining also identified primary neuron expressing increased level of synaptophysin in the group receiving 200μg/ml CPPs treatment of the Aβ pretreated cells (Figure 6D, 6E). These findings, therefore, suggested that high does CPPs treatment rescued the Aβ-induced toxicity in primary neuron.

### CPPs decreased the activity of active recombinant human β-Secretase *in vitro*

**Figure 7:**
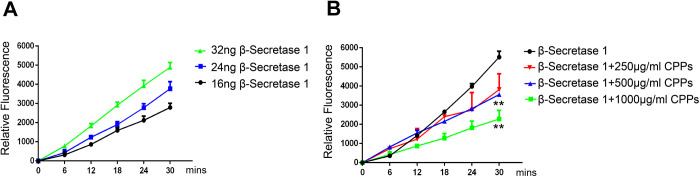
**CPPs attenuated the activity of active recombinant human β-Secretase 1 *in vitro*.** (**A**) The activity of active recombinant human β-Secretase 1 (BACE1) was measured using β -Secretase Activity Fluorometric Assay Kit. Time curves for 32ng, 24ng and 16ng β-Secretase 1 were shown. Data were from three independent experiments. (**B**) 32ng active recombinant human β-Secretase 1 and indicated concentration of 250, 500, 1000 μg/ml CPPs were pre-incubated for one hour. BACE1 activity was measured. Data were from three independent experiments. P value significance is calculated from a one-way ANOVA or two-way ANOVA. **P < 0.01 vs. β-Secretase 1 without CPPs treatment.

We have shown that CPPs attenuated Aβ toxicity and BACE1 activity in mice and in cell lines, therefore it is tempting to examine the possibility that CPPs might inhibit BACE1 activity under appropriate conditions *in vitro*, in particular at higher concentrations. To evaluate this hypothesis, we firstly measured the activity of active recombinant human BACE1 at three levels (16, 24, 32 ng) and found that its enzyme activity increased with the increase of its level (Figure 7A). Therefore, we incubated 32 ng active recombinant human BACE1 with different concentration of CPPs (250, 500, 1000 μg/ml) and measured BACE1 activity using β-Secretase Activity Fluorometric Assay Kit. The results showed that co-incubation of CPPs at 500 and 1000 μg/ml for one hour significantly decreased BACE1 activity *in vitro* (Figure 7B). The data suggested that CPPs directly inhibited BACE1 activity and therefore attenuated Aβ toxicity in APP/PS1 mice.

## DISCUSSION

Alzheimer's disease is a progressive neurodegenerative disease, and its histopathological hallmarks include abnormal neurofibrillary tangles, formed by hyperphosphorylated microtubule-associated protein Tau, and extracellular aggregates of Aβ plaques [34]. Moreover, Amyloid can induce neurofibrillary tangle formation in Alzheimer's disease mouse model, indicating that Aβ causes or enhances the tangles pathology [35]. Here APP/PS1 mice model was used to study the efficiency of CPPs treatments on AD. Using four different behavioral tests, we found that a one-month IG regiment of CPPs significantly attenuated cognitive impairments, decreased Aβ deposition in the APP/PS1 mice at the same time. We also observed slightly mitigated anxiety-like behavior of APP/PS1 mice treated with CPPs, further studies are required to analyze the pharmacological function of CPPs on anxiety. Meanwhile, the altered synaptic proteins PSD95 and synaptotagmin in the APP/PS1 mice were significantly recovered, suggesting that CPPs might alleviate AD related memory impairment by restoring synapse related proteins and thus synaptic plasticity. Moreover, the loss of dendritic spine density was also recovered by CPPs treatment.

BACE1 is a rate-limiting enzyme in the amyloidogenic pathway that cuts APP, releasing truncated APPβ and a C-terminal fragment (C99), which starts the amyloid cascade process, whereby C99 is further cleaved by γ-secretase to produce insoluble Aβ peptides [36, 37]. It has been reported that heparan sulfate polysaccharides interfere with the activity of BACE1 by interacting directly with BACE1 [23]. Interestingly, treatment CPPs, a type of polysaccharide, at a dose of 300mg/kg in APP/PS1 mice, resulted in a significant decrease in the amount of APPβ along with a decreased activity of BACE1, indicating that CPPs might ameliorate AD related symptoms by interfering BACE1 activity. In cellular assays, different concentrations of CPPs were co-incubated with N2a-APP cells and HEK293 cells transfected with APP plasmid. The results showed that the expression of APPβ was decreased at high concentrations of CPPs treatment. Most importantly, BACE1 activity was also found to be decreased, suggesting that CPPs might affect the deposition of Aβ peptides by affecting the activity of BACE1. Further *in vitro* experiments showed that CPPs with high concentration could significantly reduce the enzymatic activity of BACE1.

In the current study, the effect of CPP on Aβ clearanace haven’t been investigated. Actually, the clearance of Aβ in cell can be achieved through degrading enzymes such as Neprilysin (NEP), Insulin degrading enzyme (IDE), Endothelin converting enzyme (ECE) and Angiotensin converting enzyme (ACE), while the clearance of extracellular Aβ is mainly depended on glial phagocytosis [38]. Numerous studies have shown that microglia, the innate macrophages in central nervous system, play a vital role in the phagocytosis of Aβ. Previous studies have shown that CPPs might promote the macrophage phagocytosis and meliorate the inflammatory response in several cell and animal models [39–41]. Further researches are required to determine whether the phagocytosis of microglia is also enhanced by CPPs, thus affect the clearance of Aβ.

Several potential drugs have been produced for the treatment of AD induced cognitive deficits, and some of them had reached Phase I, II, and III clinical trials [15, 42]. However, a very few of the current therapeutic drugs were thought to be effective in reversing the development of AD. Most of the drugs are only effective in managing the symptoms but do not stop the progression of the disease. Many of the drugs showed promising results in *in-vivo* studies, but failed in human clinical trials, mainly because of the instability and less bioavailable of drugs, thus new strategies are needed urgently. Increasing number of studies have revealed that active compounds extracted from natural sources (Chinese herbal medicine) showed better biological activity and less side effects and therefore attracted attention and became promising therapeutic agent for neurodegenerative diseases [43] including AD. It has been reported that Gastrodin, a phenolic glucoside extracted from the Chinese herbal medicine Gastrodia elata Blume, has antioxidant, anti-inflammatory, and antiapoptotic effects in several cell types [44]. Moreover, Gastrodin is blood-brain barrier (BBB)-permeable, and has been proved to alleviate different stressors-induced cognition impairments in experimental animals. In the present study, our results showed that CPPs had a protective effect on APP/PS1 mice through alleviating behavioral deficits and Aβ pathology. Further studies are required to analyze the effective components of CPPs polysaccharides and whether they have ability to cross the BBB.

Previous study showed that midi-GAGR, a BBB-permeable polysaccharide, with long plasma half-life and neuroprotective properties, had protective role in coincubation with rodent cortical neurons following exposure to Aβ1-42 [24]. Accumulating evidence indicates that a group of polysaccharides have neuroprotective effects, raising the possibility of polysaccharides as candidate for the treatment of neurodegenerative diseases [45]. In this study, we also found that CPPs could protect primary neurons from Aβ1-42-induced cytotoxicity, with increased expression of synaptic proteins, and synaptic complexity. The mechanism underlying the protective effect of CPPs on primary neuron needs further investigation.

Altogether, the results from our study showed that one-month intragastric administration of CPPs attenuated cognitive impairments in APP/PS1 mice, and this effect was associated with reduced Aβ levels in the hippocampus and reduced BACE1 activity, indicating that the beneficial effects of CPPs treatment might be mediated through the suppression of BACE1 activity. Whether some molecules in the CPPs involved in the mitigation of cognitive impairments remained to be determined. Our findings had proved that as a natural herbal medicine, CPPs can be an effective therapeutic agent for the treatment of AD.

## MATERIALS AND METHODS

### Plasmids, chemicals and antibodies

Wild-type APP770 plasmid was generous gift from Prof. Angela Ho (Boston University, Boston, MA, USA). *Codonopsis pilosula* polysaccharides (purity: 92.2%) were obtained from Department of Chinese Medicine (Medical College, Hubei University for Nationalities, Enshi, China).

Antibodies used in this study are listed in Table 1.

**Table 1:** Antibodies employed in this study.

**Antibody**	**Specific**	**Cat Number**	**Type**	**Dilution**	**Source**
PSD95	PSD95 N-terminal	507	pAb	1:1000 for WB	Cell Signaling Danvers, MA, USA
synaptotagmin	Total synaptotagmin	13259	mAb	1:1000 for WB	Abcam, Cambridge, MA, USA
synaptophysin	amino acids 221-313 of SYP	17750	mAb	1:1000 for IF	Santa Cruz Biotechnology
APPβ	Anti-human sAPPβ	18957	pAb	1:1000 for WB	IBL
APP	Full length of total APP	2452S	pAb	1:1000 for WB	Cell Signaling Danvers, MA, USA
APPα	Anti-human sAPPα	11088	mAb	1:1000 for WB	IBL
BACE1	Total BACE1	5606S	pAb	1:1000 for WB	Cell Signaling Danvers, MA, USA
GAPDH	Total GAPDH	12002	mAb	1:1000 for WB	Servicebio

Mono-, monoclonal; poly-, polyclonal; WB, Western blot; IF, immunofluorescence.

### Animal studies

Male APP/PS1 mice (5.5-month-old, 23 ± 2 g) were obtained from Nanjing Biomedical Research Institute of Nanjing University. Male C57/BL6 mice (5.5-month-old, 23 ± 2 g), supplied by the Experimental Animal Central of Tongji Medical College, Huazhong University of Science and Technology. All have free access to food and water and kept in an air-conditioned room (22 ± 2°C, 12/12-h light/dark cycle). APP/PS1 mice were randomly divided into three groups as the vehicle control (saline) group, the low dose (100 mg/kg/day) CPPs group and high dose (300 mg/kg/day) CPPs group. For CPPs group, mice were treated with daily intragastric (IG) administration of 100, 300 mg/kg of CPPs for one month. The Wild-type C57 mice and APP/PS1 control groups were given IG saline as control. Mice were taken to the behavioral testing rooms at least 1h before testing began and sacrificed after the final behavioral test.

### Cell culture and transfection

HEK293-T cells and mouse neuroblastoma N2a cells with stable expression of APP(N2A-APP) cells were cultured in DMEM (Hyclone, USA) with 10% fetal bovine serum (Gibco, USA). HEK293 cells were plated at a density of 1×10^6^ cells per well in 6-cm plates. Eighteen hours post-seeding, cells were transfected with APP plasmids using Neofect DNA transfection reagent (Neofect Biological Technology, China) following manufacturer instructions.

For primary culture, hippocampus neurons were isolated from embryonic day E17 to E18 Sprague-Dawley rats and cultured as previously described [46]. Neurons were cultured in neurobasal medium (Gibco, USA) supplemented with B-27(Gibco, USA) and GlutaMAX (Gibco, USA).

### Western blotting

Brain tissue homogenates or cell lysates were obtained as previously described [28]. Protein concentration was assessed by BCA Protein Assay Kit (P0011, Beyotime). The loading proteins were subjected to SDS-PAGE, and then transferred to nitrocellulose membranes (Amersham Biosciences). Image J software (Rawak Software, Inc. Germany) was utilized to quantitatively analyze the protein bands.

### Immunocytochemistry

Primary neurons were seeded on coverslips and fixed in 4% paraformaldehyde for 15 min, permeabilized with 0.1% Triton X-100 in PBS for 15 min. Cells were incubated with antibody against synaptophysin at 4°C overnight and secondary antibodies at room temperature for 1 h, counterstained with DAPI for 5mins. Fluorescent images were captured with fluorescence microscope (LSM710, Zeiss, Germany).

### Open field test

The animals were individually placed at the same starting point in a 50x50x50 cm^3^ white acrylic box and left to search freely within it for 5 min each and this was recorded by a video tracking system (HVS Imagen, UK). The data were gathered and analyzed.

### Elevated plus maze test

Elevated Plus Maze (EPM) test was utilized to assess anxiety-like behavior [47]. The EPM test consisted of an elevated platform with two open arms and two closed arms. Mice were placed into the center of the platform facing the same open arm, and allowed to search for 5 min each. The number of entries and duration in the opened arm, as well as the duration in the center were recorded by a tracking camera.

### Novel objective recognition test

Novel object recognition (NOR) tests were performed referring to Shentu’s study [48]. In the first day, the familiar A and B objects were put in the two corners of the white acrylic box and each mouse had 5 min to search within the box. Twenty-four hours later, the B object was replaced by the C object and the mice still had 5 min to detect two objects.

### Morris water maze test

The Morris water maze (MWM) tests were performed as previously described in Huang et al [49]. Briefly, a circular pool filled with water was used for MWM and a submerged platform was hidden 1.5 cm below the water surface. The acquisition training was carried out for five consecutive days, mice were trained to find the platform within 60s. In the seventh day, the hidden platform was removed and each mouse was allowed to search for the platform in 60s. The trajectory of the mice was monitored by a video-tracking camera.

### Golgi staining

Golgi staining was carried out using the FD Rapid GolgiStain™ Kit (FD NeuroTechnologies, USA) according to the manufacturer’s protocol. In brief, brain removed from the mice were rinsed quickly using double distilled water to remove blood from the surface without perfusion or post-fixation. Tissue was immersed in the impregnation solution containing solutions A and B pre-mixed 24 hours and stored at room temperature for 3 weeks in the dark. The brains were serially cut into 100 μm sections using a vibrating microtome (Leica, VT1000S, Germany). The sections were rinsed in double distilled water, and placed in a mixture consisting of 1:1:2 of solution D, E and double distilled water respectively for 10 minutes. Then the sections were rinsed in double distilled water and dehydrated in 50%, 75%, 95% ethanol and absolute ethanol. The sections were cleaned in xylene and covered with Permount TM Mounting Medium. Images were observed under the microscope (Nikon, Tokyo, Japan).

### β-Secretase activity assay

β-Secretase Activity Fluorometric Assay Kit (K360-100, Biovision, USA) was utilized to detect β-secretase activity following the manufacturer’s instructions. Briefly, ice-cold Extraction Buffer was added to sample and homogenized on ice. The homogenate was incubated on ice for 10 minutes and centrifuged at 10,000g for 5 minutes. The protein concentration in supernatant was measured by BCA Protein Assay Kit (Beyotime, China). A total of 200μg protein was used for the assay and the volume was adjusted to 50μl, and was then added to a 96-well plate, and 50μl Reaction Buffer was added and pre-incubated for 20 min at 37°C. Then 2 μl of β-Secretase substrate was added to the wells and the plate was covered, well mixed, and incubated in the dark at 37°C for 1 hour. The samples were read in a fluorescent plate reader with Ex/Em = 345/500 nm. For in vitro tests, 32ng active recombinant human β-Secretase 1(7609, Biovision, USA) was pre-incubated with different concentrations of CPPs in a 96-well plate in the dark (37°C, 60 min), followed by β-secretase activity test.

### ELISA quantification of Aβ40 and Aβ42

Aβ40 and Aβ42 were quantified using the Human/Mouse Aβ1-40 or Aβ1-42 ELISA Kit (Elabscience, China) in accordance with the manufacturer’s instructions. In brief, the hippocampus from mouse brains or cell suspension were homogenized in buffer (PBS supplemented with protease inhibitor cocktail) on ice and centrifuged at 2000 g for 20 min, the supernatant was collected and added to the provided micro ELISA plate coated with the anti-Aβ antibody, incubated for 90mins. Liquid in the plate was discarded and the plate was incubated with Biotinylated Detection antibody working solution for 1 hour at 37°C. The wells were washed then incubated with HRP Conjugate working solution for 30 min, again washed and incubated with a substrate reagent for 15 min at 37°C and finally the stop solution was added. The optical density was measured using micro-plate reader at a wavelength of 450 nm.

### Cell viability assay

The viability of cells treated with CPPs was determined by the CCK-8 cell viability assay kit (Dojindo, Japan). Cells were seeded at a density of 1x10^6^ cells/ml in a 96-well plate and incubated at 37°C. After indicated concentrations of CPPs treatment for 24 h, the medium was replaced with 110 μL of the fresh medium, containing 10% CCK8 reagent. After half an hour, the optical density was measured using micro-plate reader at 450 nm.

### Statistical analysis

All data were expressed as means ± SEM. Data was analyzed using GraphPad Prism 6 (GraphPad Software, La Jolla California USA, https://www.graphpad.com/). Differences between groups were assessed using one-way ANOVA or two-way ANOVA, or student’s t-test. In all cases, a value of P<0.05 was considered statistically significant.

### Ethics

No humans were used in this research. All animal experiments were approved by the Animal Care and Use Committee of Huazhong University of Science and Technology, and performed in compliance with the National Institutes of Health Guide for the Care and Use of Laboratory Animals.
